# Safety and Efficacy of Ferric Carboxymaltose for Iron Deficiency Anemia in Inflammatory Bowel Disease: A Systematic Review

**DOI:** 10.7759/cureus.76065

**Published:** 2024-12-20

**Authors:** Aparna Malireddi, Mahlet Abera, Suchith B Suresh, Mehwish Ansar, Sruthi Boddeti, Khutaija Noor, Safeera Khan

**Affiliations:** 1 Internal Medicine, Andhra Medical College, Visakhapatnam, IND; 2 Internal Medicine, Saint Paul Millennium Medical College, Addis Ababa, ETH; 3 Internal Medicine, Montefiore St. Luke's Cornwall, Newburgh, USA; 4 General Surgery, Wirral University Teaching Hospital, Wirral, GBR; 5 General Surgery, California Institute of Behavioral Neurosciences and Psychology, Fairfield, USA; 6 Obstetrics and Gynecology, Tirumala Jyothi Hospital, Visakhapatnam, USA; 7 Foundation of Clinical Research, Harvard Medical School, Boston, USA; 8 Neuropsychiatry, PsychCare Consultant Research, Saint Louis, USA; 9 Internal Medicine, Shadan Institute of Medical Sciences, Peeramcheru, IND; 10 Internal Medicine, California Institute of Behavioral Neurosciences and Psychology, Fairfield, USA

**Keywords:** ferric carboxymaltose, hemoglobin, hypophosphatemia, inflammatory bowel disease, iron deficiency anemia

## Abstract

Ulcerative colitis and Crohn's disease, two types of inflammatory bowel disease (IBD), often cause anemia, primarily due to iron deficiency and chronic inflammation. Anemia negatively affects patients' daily functioning and quality of life, causing symptoms including headaches, exhaustion, and dyspnea. In IBD, iron deficiency arises from reduced intake, chronic blood loss, and impaired absorption. While oral iron supplements are commonly used, their poor absorption and gastrointestinal side effects limit their effectiveness, especially in IBD patients. The European Crohn's and Colitis Organization (ECCO) recommends intravenous iron, such as ferric carboxymaltose (FCM), as iron deficiency anemia in IBD can be managed using a safe and efficient substitute. With regard to treating iron deficiency anemia in patients with IBD, the purpose of this study is to investigate the safety and effectiveness of intravenous ferric carboxymaltose. We conducted a thorough search of medical databases, such as the Cochrane library, PubMed, and ResearchGate, to gather relevant literature. Using the databases, we found a total of 297 relevant articles. The identified studies have been screened, eligibility criteria have been introduced, and 14 research studies were selected for inclusion. This review adhered to the Preferred Reporting Items for Systematic Review and Meta-Analysis (PRISMA) guidelines, with quality assessments conducted using the Cochrane risk of bias 2 scale for randomized tests and the Newcastle-Ottawa scale for observational examination. We reviewed 14 research articles involving 2,493 patients. Among these, five were randomized controlled trials (RCTs), and the remaining nine were observational studies. The primary outcomes assessed were the therapeutic response (defined as hemoglobin ≥2 g/dL rise or normalization, improvement in iron profile parameters) and any adverse effects after FCM is administered to IBD patients. FCM was found to be highly effective in improving hemoglobin and iron profile parameters, with a generally good safety profile. Ferric carboxymaltose was the most efficient and well-tolerated intravenous (IV) iron formulation, proving safer and more effective than other iron therapies in patients suffering from IBD. However, severe hypophosphatemia can lead to serious complications, including heart failure, pulmonary failure, rhabdomyolysis, fractures, and osteomalacia, which may worsen its long-term impact. Therefore, the risk of hypophosphatemia associated with prolonged FCM use requires careful monitoring and further research to ensure its long-term safety and assess its effects on patients' quality of life.

## Introduction and background

Two forms of inflammatory bowel disease (IBD) that are distinguished by persistent, recurrent, and intermittent inflammation of the gastrointestinal (GI) tract are Crohn's disease (CD) and ulcerative colitis (UC) [1]. Iron deficiency anemia (IDA) and anemia of chronic inflammation are among the most frequent causes of anemia, which is the most common extraintestinal sign of inflammatory bowel disease (IBD) [2]. The World Health Organization (WHO) generally defines anemia as a disorder when hemoglobin (Hb) is below average (13 g/dL in males and less than 12 g/dL in females) [3]. An iron deficiency adversely affects a patient's quality of life (QoL), capacity for work, and daily functioning. Fatigue, headaches, dizziness, tachycardia, shortness of breath, diminished cognitive function, and depression are among the symptoms that are linked to it [4,5].

Reduced iron intake from dietary limitations, continuous loss of blood from the ulcerated intestinal mucosa, as well as a lack of transluminal iron absorption during disease activity, are the main causes of iron insufficiency in IBD patients [6]. Low serum iron levels can result from impaired erythropoiesis and iron metabolism due to elevated serum hepcidin levels linked to high systemic inflammation. This can also impede dietary iron absorption [7].

In patients with IDA in IBD, supplementing iron is recommended to normalize Hb levels and replenish the iron stores [8]. To prevent recurrent anemia and maintain iron reserves, raising serum ferritin (s-ferritin), transferrin saturation (TSAT), and hemoglobin levels over the lower threshold of normal is the goal of IDA treatment [6]. Iron replacement with oral iron preparations is the standard treatment for iron deficiency anemia (IDA). However, there may be restrictions on how these drugs are used due to poor absorption and gastrointestinal side effects, especially in patients with IBD [7]. Up to 90% of iron that is taken orally is not absorbed and is oxidized in the stomach, producing reactive oxygen species that can damage mucosa and have negative effects on the gastrointestinal tract [9]. Due to this risk, some nations do not recommend oral ferrous compounds for patients with IBD, and there may be limited compliance even among patients who meet the requirements for oral iron administration therapy [4]. This highlights the need for alternative treatment options that are both effective and better tolerated, leading to the consideration of intravenous (IV) iron formulations for managing iron deficiency anemia in patients with IBD.

Thus, recommendations from the European Crohn's and Colitis Organization (ECCO) advise intravenous (IV) iron as the first-line treatment in patients with clinically active IBD, a history of intolerance to oral iron, and hemoglobin levels below 10 g/dL [2]. A variety of IV iron formulations are available to treat IDA. These formulations can be classified as labile, semi-labile, or stable iron complexes based on their complex chemistry [10]. Iron polymaltose (IP), iron sucrose (IS), and ferric carboxymaltose (FCM) are a few of the most modern IV iron preparations [11].

The molecular structure of FCM contains stable iron in the form of a non-dextran iron complex with an iron hydroxide core (III) and a carbohydrate ligand. Iron can be absorbed without the body releasing free iron because of its similar structure to ferritin [12]. Due to its minimal immunogenic profile, this non-dextran IV iron treatment is less likely to result in anaphylactic responses. Administration is safe and clinically well-tolerated even at high dosages (maximum dose of up to 1000 milligrams) because of these pharmacological features [13].

Our goal in conducting this systematic review is to examine the effectiveness and safety of IV ferric carboxymaltose in treating iron deficiency anemia in individuals with IBD.

## Review

Methods

Methodology

The Preferred Reporting Items for Systematic Review and Meta-Analysis (PRISMA) 2020 standards have been adhered to in the execution of this review [14]. All included studies were evaluated against the PRISMA criteria to assess their quality, transparency, and adherence to reporting standards, ensuring that our review met the highest level of systematic review methodology.

Search Sources and Strategy

We searched PubMed, the Cochrane library, and ResearchGate to search for studies evaluating the effectiveness of FCM in patients suffering from IBD and iron-deficient anemia. Using various combinations of ferric carboxymaltose, iron deficiency anemia, and IBD, we searched every database. Finding studies that evaluated the safety and/or effectiveness of FCM administration in the management of IDA in individuals with IBD was the main goal. The use of the specific terms "ferric carboxymaltose," "iron deficiency anemia," and "inflammatory bowel disease" for the literature review is justified as these terms precisely capture the key elements of the topic. FCM is the IV iron formulation under investigation; iron deficiency anemia is a frequent complication in patients with IBD, and the combination of these terms ensures a focused and relevant search in databases, yielding studies that directly address FCM's safety and effectiveness in this particular patient population.The search strategy utilized for the Cochrane library, PubMed, and ResearchGate databases included the following key terms: ferric carboxymaltose, IDA, and IBD.

However, in addition to these keywords, the following strategy was created and applied in PubMed to search the MeSH database: ("Anemia, Iron-Deficiency/drug therapy"[Majr] OR "Anemia, Iron-Deficiency/prevention and control"[Majr] OR "Anemia, Iron-Deficiency/therapy"[Majr]) AND ("Inflammatory Bowel Diseases/complications"[Majr] OR "Inflammatory Bowel Diseases/drug therapy"[Majr] OR "Inflammatory Bowel Diseases/prevention and control"[Majr]) AND ("ferric carboxymaltose" [Supplementary Concept]) AND ("Ferric Compounds/adverse effects"[Majr] OR "Ferric Compounds/therapeutic use"[Majr] OR "Ferric Compounds/toxicity"[Majr]).

The following strategies have been made to search the PubMed advanced search builder: (((Ferric carboxymaltose[Title/Abstract]) AND (Iron deficiency anemia[Title/Abstract])) AND (Inflammatory bowel disease[Title/Abstract]) AND (("2009"[Date - Publication]: "2024"[Date - Publication]))

Table 1 lists the databases that were used along with the number of papers found in each database.

**Table 1:** Keywords/strategy used and the number of identified papers.

Keywords/Search strategy	Database used	Number of papers identified
Ferric carboxymaltose AND Iron deficiency anemia AND Inflammatory bowel disease	Cochrane Library	53
( "Anemia, Iron-Deficiency/drug therapy"[Majr] OR "Anemia, Iron-Deficiency/prevention and control "[Majr] OR "Anemia, Iron-Deficiency/therapy"[Majr ) AND ("Inflammatory Bowel Diseases/complications"[Majr] OR "Inflammatory Bowel Diseases/drug therapy"[Majr] OR "Inflammatory Bowel Diseases/prevention and control"[Majr ) AND ("ferric carboxymaltose" [Supplementary Concept]) AND ("Ferric Compounds/adverse effects"[Majr] OR "Ferric Compounds/therapeutic use"[Majr] OR "Ferric Compounds/toxicity"[Majr])	PubMed (MeSH)	28
Ferric carboxymaltose AND Iron deficiency anemia AND Inflammatory bowel disease	Pubmed	82
(((Ferric carboxymaltose[Title/Abstract]) AND (Iron deficiency anemia[Title/Abstract])) AND (Inflammatory bowel disease[Title/Abstract])) AND (("2009"[Date - Publication] : "2024"[Date - Publication]))	Pubmed (adv)	41
Ferric carboxymaltose AND “Iron deficiency anemia AND Inflammatory bowel disease	Research Gate	93
Total number of papers identified		297
Number of articles after removing duplicates		162

*Inclusion and Exclusion Criteria* 

The following were the inclusion criteria for this review: adults (aged 18 years and above) having a confirmed diagnosis of IDA in the context of IBD, irrespective of gender or ethnicity; the intervention involved intravenous ferric carboxymaltose, with no restrictions on dosage or duration, compared to either placebo or another intervention in the control group; the primary outcome indicator has been the proportion of patients who experienced a hematopoietic response, which was described as either a return to baseline hemoglobin (Hb) levels or an increase of at least 2 g/dL, additionally, improvements in iron profile parameters, like serum ferritin, transferrin saturation (TSAT), serum iron, and mean corpuscular volume (MCV), were assessed and the overall frequency of major adverse events and drug-related adverse events was evaluated by the secondary outcome measure; study design included studies that were randomized controlled tests (RCTs), clinical trials, observational research (both prospective and retrospective), cohort studies, and multicenter studies, regardless of sample size, published in English over the last 15 years (2009-2024).

The exclusion criteria were clearly defined to maintain the integrity of the review: studies involving children or adolescents; animal studies and studies without full-text availability; gray literature, to maintain the focus on peer-reviewed, high-quality evidence; reviews, meta-analyses, editorials, systematic reviews, conference abstracts, and posters. This exclusion ensured the review focused solely on original research with direct clinical data, allowing for the inclusion of high-quality, comprehensive data on the safety and effectiveness of injecting FCM intravenously to treat IDA in IBD patients.

 *Screening Process*

The duplicate articles were eliminated, and the shortlisted articles were transferred to EndNote (Clarivate, London, UK). Titles and abstracts were used to filter the articles. Only pertinent articles were examined, and the shortlisted articles were further analyzed based on their full content. Only the articles that met the requirements were shortlisted after the inclusion and exclusion criteria were applied.

Quality Assessment of the Studies

The shortlisted articles were evaluated using the relevant quality assessment instruments. The Newcastle-Ottawa instrument was employed to assess the caliber of observational research. The Cochrane risk of bias assessment tool was used to evaluate the bias risk of RCTs. The following domains guided its conduct: random sequence creation (selection bias), any other bias that can be identified, such as allocation concealment (selection bias), personnel and participant blinding (performance bias), outcome assessment blinding (detection bias), incomplete outcome data (attrition bias), selective reporting (reporting bias), and so on. Inclusion in the systematic review was restricted to studies that met the quality appraisal criteria.

Data Collection Process

Following the extraction and finalization of the papers for the systematic review, the primary findings were assessed in conjunction with additional pertinent information. The following information was taken from each study and tabulated: author/year of publication, type of study, drug intervention, number of participants, duration of the study, efficacy and safety outcomes, and conclusion of the study [15]. Finalizing the received data involved equal participation from all writers.

Results

Study Identification and Selection

Using the databases, we found a total of 297 articles that were relevant. Before screening, a total of 135 duplicate articles were removed. Thirty-one articles were shortlisted after these were screened by looking at the titles, abstracts, and complete texts. After evaluating these articles for quality and suitability, 14 of them were selected for review.

**Figure 1:**
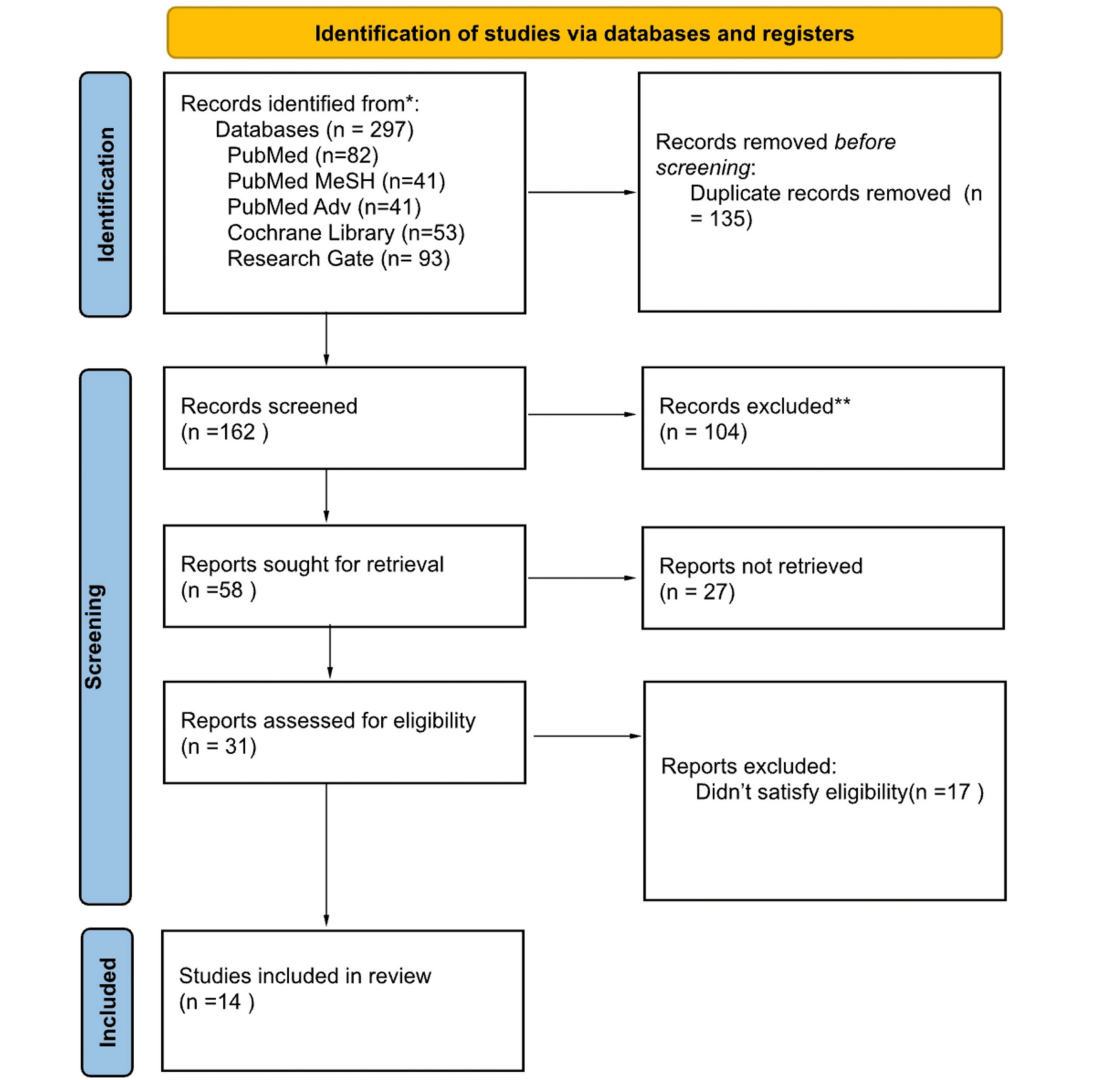
PRISMA flowchart showing the process of article selection. PRISMA: Preferred Reporting Items for Systematic Review and Meta-Analysis

Figure 1 presents the PRISMA flow chart, illustrating the study selection process.

Quality Appraisal

The articles' eligibility was evaluated utilizing the appropriate quality appraisal instruments. The quality appraisal findings are displayed in the table below.

Table 2 displays the findings of the quality appraisal of the observational studies. Table 3 displays the findings of the quality appraisal of the randomized controlled trials. 

**Table 2:** Quality appraisal of the observational studies by the Newcastle-Ottawa scale.

Study by/year of publication	Selection	Comparability	Outcome	Overall
Befrits et al. 2013 [16]	***	*	***	7/9
Siqueira et al. 2023 [12]	***	*	**	6/9
García-López et al. 2016 [17]	***	*	***	7/9
Kangaspunta et al. 2018 [18]	***	*	***	7/9
Beigel et al. 2012 [19]	***	*	***	7/9
Stein et al. 2018 [6]	***	*	***	7/9
Cortes et al. 2015 [20]	****	**	***	9/9
Sobrado et al. 2015 [21]	***	*	***	7/9
Detlie et al. 2021 [22]	****	*	***	8/9

**Table 3:** Quality appraisal of the randomized controlled trials by the Cochrane bias assessment tool.

Study by/year of publication	Selection bias	Allocation concealment	Performance bias	Detection bias	Attrition bias	Reporting bias	Overall
Howaldt et al. 2022 [4]	low risk	low risk	unclear risk	unclear risk	unclear risk	low risk	unclear risk
Evstatiev et al. 2011 [23]	low risk	low risk	unclear risk	unclear risk	low risk	low risk	unclear risk
Evstatiev et al. 2012 [24]	low risk	low risk	low risk	unclear risk	low risk	low risk	low risk
Bertani et al. 2021 [25]	low risk	low risk	unclear risk	unclear risk	low risk	low risk	unclear risk
Zoller et al. 2022 [26]	low risk	low risk	low risk	low risk	low risk	low risk	low risk

Study Characteristics

We reviewed a total of 14 research articles comprising 2,493 patients. This included five randomized controlled trials (RCTs) with a combined total of 1,281 participants and nine observational studies with 1,212 participants. Among the RCTs, the trials conducted by Evstatiev et al. (2011) and Bertani et al. both had a duration of 12 weeks, Zoller et al. spanned 10 weeks, and Howaldt et al. extended over 52 weeks. Bertani et al. and Howaldt et al. compared FCM with oral iron preparations, specifically microsomal iron and ferric maltol, respectively. Zoller et al. primarily focused on the safety profile of FCM [4,23,24,25,26].

Among the nine observational studies, four were retrospective, while the others were prospective in nature. The number of participants enrolled ranged between 10 [21] and 483 [23]. All studies included patients with IBD and IDA and reported on efficacy outcomes (such as increased hemoglobin and improved iron parameters), safety outcomes (such as adverse effects following FCM treatment), or both. Notably, a particular study looked at the safety of giving infliximab and FCM together in a single session. Three studies (Detlie et al., Zoller et al., Evstatiev et al., 2011) made a straight comparison between two IV iron formulations.

Table 4 shows the summary and characteristics of all the included studies.

**Table 4:** Shows the summary and  characteristics of all the included studies. FGF23: fibroblast growth factor 23; FDI: ferric derisomaltose, IFX: Infliximab; IDA: iron deficiency anemia; IS: iron sucrose; IBD: inflammatory bowel disease; FCM: ferric carboxymaltose; AE: adverse events; UC: ulcerative colitis; CDAI: Crohn's disease activity index; CRP: C-reactive protein; CD: Crohn’s disease; Hb: hemoglobin; MCV: mean corpuscular volume; TEAE: treatment associated adverse effect; ALT: alanine aminotransferase; TSAT: transferrin saturation; IV: intravenous; SI: sucrosomial iron

Study(year)	Types of study	Drug intervention	Number of participants	Duration of study	Efficacy outcomes	Safety outcomes	Conclusion
Befrits et al. 2013 [16]	Prospective observational study	IV, FCM; median initial dose 1000 (100-1000)mg	394 (group A-216, group B-178)	17 (13–29) months for group A, 12 months for group B	The median values of Hb, ferritin, and T-Sat following iron treatment were 134 g/l, 121 mmol/l, and 20%, respectively, compared to baseline values of 111 g/l, 10 mmol/l, and 10% (p < 0.001 for all three parameters). Similar results were noted for group B.	Twenty-seven individuals (7%) reported infusion reactions. The most frequent ones were pruritus, nausea, chest oppression, and elevated body temperature. None of them were considered significant.	Over time, there was an improvement in the adherence to European guidelines. Hemoglobin, T-Sat, and ferritin levels were significantly increased following therapy, according to the overall results. Almost 75 per cent of the individuals experienced the effect for more than a year.
Siqueira et al. 2023 [12]	Retrospective observational study	IV, FCM; single dose (500mg)	25	-	93% of patients had elevated Hb levels during FCM treatment, and the median Hb concentration rose from 8.5 g/dL to 10.1 g/dL (P < 0.0001). The median concentration of hematocrit increased significantly in 88% of patients, going from 27.8% to 33%(P < 0.0001). The median serum iron improved in 18 patients (86%) from 15 µg/dL to 26 µg/dL (P < 0.0001). Ferritin levels increased in 86% of patients after FCM, going from 23.79 ng/mL at baseline to 100.38 ng/mL at the end of treatment. Following therapy, this parameter normalized in 77% of patients (P = 0.0008). TSAT raised from 3.5 at the start of the regimen to 9 following FCM injection (P = 0.01).	No information about safety was reported.	The study demonstrated the significance of FCM as a therapeutic approach by demonstrating its superiority to iron hydroxide sucrose delivery in the treatment of individuals with refractory IDA.
Gracia- Lopez et al. 2016 [17]	Prospective cohort study	IV, FCM, the Ganzoni formula was used to compute the total iron dose, which was 1 or 2 infusions spaced 7 days apart with a maximum of 1 g (or 20 mg per kg) each infusion	72	12 weeks	At week two, 42% of the therapy courses (88 treatment courses given to 72 participants) had produced a response. At week 12, the response was obtained with full follow-up in 81.2% of treatment courses.	There was only one adverse event (AE) (hypertension, minor chest discomfort, dyspnea, and flushing) that was thought to be associated with FCM. With the use of IV fluids and a prompt end to the infusion, symptoms were brought under control without further clinical consequences.	In conclusion, high-dose FCM infusion proved to be both very safe and successful in treating IBD anemia in clinical settings. It had a very quick beginning the action; by week 2, a significant number of patients had fully recovered from their anemia, and their quality of life had significantly improved. There was no correlation between treatment and increased biological or clinical IBD activity.
Beigel et al. 2012 [19]	Retrospective cohort study	IV, FCM (range 100–7,500 mg), median dose 500 mg	250	20 weeks	90.7% of the follow-up participants reached Hb levels > 12/13 g/dl (p<0.01), 61.6% of the participants reached the ferritin goal value of >100 ng/ml (p < 0.001), and 74.6% of the participants reached the goal serum iron level of >60 μ g/dl (p<0.001). These results were compared to pre-treatment values.	Therapy-related AEs occurred in 17.5% of patients; the most frequent was a temporary elevation of liver enzymes (ALT and/or APT) that was more than twice the upper limit (15.0%) and totally disappeared at follow-up. A headache and edema in the feet are two more.	According to this study, the majority of IBD patients can return to normal anemia with just a single 500 mg dose of FCM, making it an incredibly effective and well-tolerated medication for IBD patients.
Sobrado et al. 2015 [21]	Prospective study	IV, FCM: average dose of 1650 (1500–2000) mg	10	12 weeks	Eight patients had a Hb rise of at least two points following intravenous FCM infusion, and anemia normalization occurred in seven patients (70%).	Only three patients experienced moderate AE (two with nausea and one with headache), which resolved on their own without the need for intervention or withdrawal of the medication.FCM was well tolerated.	The present study shows a strong association between the severity of anemia and the level of CD activity as determined by CRP and CDAI. Ferric carboxymaltose is a safe and quick way to treat anemia in people with CD while also improving their quality of life.
Kangaspunta et al. 2018 [18]	Retrospective observational study	IV, FCM, average dose 1050 mg	87	-	After six months, there was an average increase in hemoglobin levels of 26.0 g/l with SD ±19.0 g/l (n = 82) and an increase in MCV of 9.28 ± 7.14 fl (n = 83). No statistically significant differences in the therapeutic response to intravenous iron therapy between patients with CD or UC in terms of hemoglobin levels or MCV was observed.	During the iron infusion, 10.3% of individuals experienced the following adverse events: hematoma, nausea , dizziness, fainting, rash (2), and vein irritation (3).	This study concludes FCM to be a safe and effective method to treat severe anemia as well as during active disease. The effectiveness of the treatment could be assessed through plasma ferritin and transferrin receptor concentrations.
Stein et al. 2018 [6]	Prospective observational study	IV, FCM, mean 1139mg (100-4800 mg)	223	12 weeks	63% per cent of patients achieved the primary efficacy endpoint, which was Hb normalization or a rise of ≥2 g/dL. After receiving FCM treatment, the mean ferritin level in the efficacy group increased to 110 (±148) μg/L from 53 (±126) μg/L; mean TSAT level increased from 15.6 (±13.4)% to 24.2 [±20.2]% (p <0.001).	The safety population consisted of 193 individuals, and just one adverse event—an exacerbation of UC was documented, although it was unrelated to the medication.	This study demonstrated FCM medication to be safe and effective in routine practice. It minimizes patients' missed work time, at the same time is much more convenient for the physician due to rapid, large dose administration.
Detlie et al. 2021 [22]	Prospective observational study	IV, FCM, 1000 mg; IV, FDI, 1000 mg	106 (FCM=52, FDI=54)	7 weeks	No information about efficacy was reported.	After beginning FCM treatment, at weeks two and six, hypophosphatemia was detected in 72.5% and 21.6% of the patients, respectively. During weeks two and six of the FDI therapy group, hypophosphatemia was reported by 3.7 percent and 11.3 percent of patients, respectively. At weeks two and six, the difference in incidence was statistically significant (P < 0.001 and P < 0.013, respectively). The patients receiving FCM showed a substantial rise in mean concentrations of intact fibroblast growth factor 23 (P < 0.001), a significant drop in 1,25-dihydroxyvitamin D and ionized calcium levels, and a significant increase in mean urine fractional excretion of phosphate (P = 0.004).	The study concluded that fibroblast growth factor 23 plays a major part in FCM-induced hypophosphatemia, most likely by causing phosphate loss through urine.
Evstatiev et al. 2011 [23]	RCT	IV, FCM, (mean total dose: 1377 ± 381 mg); IV, Iron sucrose IS, (mean total dose: 1160 ± 316 mg)	483 (FCM subgroup; 244 IS subgroup: 239)	12 weeks	A non-anemic state or a Hb rise of ≥2 g/dL was more likely to be achieved by the FCM group than by the IS group (191 (83.8%) vs. 167 (75.9%) of patients; odds ratio (OR), 1.67; 95% CI: 1.04–2.67; P=.033), according to the data. Repeated measures analysis showed that the FCM group had significantly higher increases in ferritin (at all time periods), TSAT, and Hb (beginning in week 2).	The common AEs in the FCM group include: hyperferritinemia(7), hypophosphatemia(6), rash(4), headache (3), iron overload, infusion site reactions, withdrawn because of adverse event (7), severe adverse (pulmonary embolism)-1. Under FCM, the mean serum phosphate levels dropped from baseline to week 2, normalized between weeks, 4 and 12. The frequency of adverse events attributable to drugs was similar in both groups (P = 0.413).	The study shows that the new, simple fixed-dose FCM regimen which included up to three infusions and was based on weight and baseline hemoglobin levels proved to be more effective than the Ganzoni- calculated IS dosage currently employed in clinical settings.
Evstatiev et al. 2012 [24]	RCT	IV, FCM, 1000 mg; placebo- saline	245 (FCM-105, placebo-99, no treatment- 41)	-	According to a Kaplan–Meier study, patients receiving FCM had a far lower chance of developing anemia (27.2% vs. 40.4%) than those receiving a placebo. The serum ferritin level and TSAT improved in FCM-treated patients (+30.3 μg/L and +0.58%) but not in placebo-treated patients (−36.1 μg/L and −4.00%).	Treatment associated adverse effect (TEAE) were observed for 50.5% (placebo) and 59.0% (FCM) of patients, whereas 6.7% and 8.1% of patients experienced significant AEs. The most frequent TEAEs were nasopharyngitis (7.3%) and symptoms related to ulcerative colitis (UC) (7.8%).	Patients whose anemia has responded to IV iron replacement therapy in the past can benefit from safe and efficient treatment with serum ferritin-triggered therapy in conjunction with FCM. It is necessary to make adjustments to the treatment trigger, FCM dose, and dosing interval in addition to identifying high-risk populations.
Bertani et al. 2021 [25]	RCT	IV, FCM 1000 mg, one infusion; oral sucrosomial iron 60 mg q.d. for 8 weeks since baseline and then 30 mg q.d. for 4 weeks	40 (FCM-20, SI- 20)	12 weeks	Following a four-week course of therapy, blood Hb levels rose by an average of +11% in both groups and remained stable during follow-up. Treatment and treatment-by-time interaction effects were not statistically significant (p = 0.25 and 0.46, respectively). After a four-week course of treatment, both groups' blood hemoglobin levels increased by an average of +11% and stayed steady during the follow-up. The interaction effects between treatment and treatment-by-time (p = 0.25 and 0.46, respectively) did not reach statistical significance. After four weeks of either therapy, serum iron levels rose by an average of +278% and then peaked. There were no significant treatment differences (treatment effect, p = 0.25; treatment-by-time interaction effect, p = 0.26).	Both treatments were well tolerated. Oral iron supplementation was linked to a patient's temporary moderate gastrointestinal symptoms (flatulence and bloating) (5% vs. 0% incidence in the FCM group, p > 0.99),	This study demonstrated that SI and FCM are equally useful in treating anemia in UC patients who are in remission, therefore suggests the use of oral iron supplementation in these patients because of its higher patient acceptance, reduced cost, and effectiveness and tolerability.
Zoller et al. 2022 [26]	RCT	IV, FCM-1000 mg ; IV, ferric derisomaltose- 1000 mg	97 (FCM-49, FDI- 48)	10 weeks	By day 70, ferritin and TSAT increased similarly in the FCM and FDI treatment groups, producing significant increases in hemoglobin levels (FDI:24.9g/L; FCM: 25.2g/L).	At every visit, the frequency of hypophosphatemia was greater in patients treated with FCM than in patients treated with FDI; the incidence peaked two weeks following the first FCM therapy at 45.8%. Most patients recovered from hypophosphatemia by day 70; however, 4.7% of FCM-treated patients persisted in being hypophosphatemic for more than a month following the second infusion, and there was a significant difference in the mean serum phosphate levels between FCM-treated and FDI-treated patients on day 70. When compared to FDI, intact FGF23 concentrations increased significantly following both FCM infusions. Phosphate excretion in the urine was greater in patients receiving FCM.	This trial demonstrated that hypophosphatemia is a relatively prevalent side effect of FCM, driven by significant elevations in FGF23 rather than a class or dose impact of intravenous iron. Even with similar dose schedules, FDI has far less of an impact on FGF23 and serum phosphate.
Cortes et al. 2015 [20]	Prospective observational study	Single-session group patients received FCM after Infliximab( IFX) on the same day, control group patients received FCM on a separate day from IFX. All patients received 5mg/kg IFX and 1000mg FCM for iron-restricted anemia (IRA) or 500 mg FCM for iron deficiency without anemia	45 (Single-session group patients (n = 33), Control group patients (n = 12))	8 weeks	At week 8, 79% and 67% have Hb increase ≥2 g/dL or normalized Hb levels or normalized TSAT or s-ferritin in the single session group patients and control group patients respectively. The mean cost of administration per patient was 354.63€ and 531.94€ in the single session group patients and control group patients, respectively. No statistically significant differences were observed between the control and single-session treatment groups for any of these efficacy metrics.	Regardless of whether the IV administration of both medications occurred on different days or in a single session, no adverse events (AEs) were reported at the end of the IV administration (both IFX and FCM) in either group (control or single session).	The study shows that this single-session regimen may offer a better cost-benefit ratio than the standard clinical practice of injecting IFX and iron on different days while also enhancing therapy adherence and patient quality of life.
Howaldt et al. 2022 [4]	RCT	IV FCM is given according to each center’s standard practice. Oral ferric maltol 30 mg twice daily	416 intention to treat (ITT)- 250 (ferric maltol- 125 / FCM- 125) PP (per protocol)- 166 (ferric maltol-78 /FCM-88)	52 weeks	In the intent-to-treat and per-protocol analyses, the responder rates at week 12 were 67% and 68%, respectively, for ferric maltol and 84% and 85%, respectively, for FCM.The primary endpoint hemoglobin responder rate (≥2g/dL increase or normalization) was not reached since the confidence intervals went over the non inferiority margin.	TEAE occurred in 59% and 36% of patients receiving ferric maltol vs. FCM respectively. These were mainly gastrointestinal in the ferric maltol group and infections/infestations in the FCM group. The most commonly reported were upper abdominal pain and nausea.	Over the course of a 12-week treatment, both oral ferric maltol and conventional IV FCM regimens produced clinically significant increases in hemoglobin. However, ferric maltol did not satisfy the predetermined noninferiority margin when compared to IV iron. Up to week 52, ferric maltol showed comparable long-term efficacy in maintaining hemoglobin increases and elevating ferritin. According to the study's findings, ferric maltol provides IBD patients with a straightforward, long-term treatment for chronic IDA.

Discussion

While there are many reasons for anemia in people with IBD, iron deficiency is by far the most common etiology [15]. IDA is caused by a variety of factors, such as hemolysis, malnutrition, and chronic blood loss [27,28]. Even in cases when anemia is not overtly symptomatic, it nevertheless has a major impact on the QoL for patients with IBD. Therefore, it's critical to manage anemia efficiently and avoid assuming that a degree of anemia is a typical aspect of IBD [28].

Oral iron was initially the treatment of choice; however, it was demonstrated to impact the composition of the microbiome [29], which is crucial to the pathophysiology of IBD [30]. In contrast to compounds like high molecular weight (HMW) iron dextran, which were linked to serious safety issues, more recent IV iron compounds like iron sucrose, FCM, and iron isomaltoside have been demonstrated to be both generally safe and efficacious [15]. Moreover, it has been shown that intravenous iron administration results in a quick and more efficient replenishment of the body's iron reserves compared to oral iron supplementation [31]. This superiority of IV iron over oral iron has already been demonstrated in some meta-analyses [32,33]. 

This study sought to examine, compare, and summarize data from printed trials and observational studies evaluating the safety and efficacy of intravenous ferric carboxymaltose in the therapy of IDA in patients having IBD. The goal was to enhance the body of evidence supporting the use of this contemporary intravenous iron therapy.

Efficacy of Intravenous Ferric Carboxymaltose

The efficacy of FCM in managing IDA among IBD patients was well-established in multiple clinical trials and observational studies. These studies consistently report significant improvements in hemoglobin (Hb) levels and iron profile parameters such as serum ferritin and transferrin saturation (TSAT) after FCM administration. A notable advantage of FCM is its ability to restore iron levels and improve anemia-related symptoms quickly, often with a single or a limited number of infusions. For example, García-López et al. conducted a study in which 72 patients received intravenous FCM (maximum of 1000 mg per infusion) and reported a response in 81.2% of treatment courses in a short duration of three months. FCM had a very quick beginning of the action; by week two, a significant number of patients had fully recovered from their anemia, and their quality of life had significantly improved [17].

The efficacy of ferric carboxymaltose on increasing hemoglobin was evaluated in a prospective study in 2013 by Befrits et al. in Swedish patients over 17 months, which found that a median dose of 1000 mg of intravenous ferric carboxymaltose increased hemoglobin of ‡20 g/l in 45% of patients; 75% of patients achieved a normal hemoglobin level; and an increase in ferritin level in 52% of patients (p < 0.001 for all three parameters). The study reported an improved adherence to the European guidelines and concluded that about 75% of the individuals experienced the effect for more than a year [16]. A retrospective study by Kangaspunta et al. showed that over the course of six months, approximately 94% of the patients experienced a similar and significant improvement in their hemoglobin and MCV value; however, there were no statistically significant variations in the therapeutic response between patients with CD and UC [18]. A retrospective study done by Stein et al. showed that Ferinject used in routine practice was both safe and effective. After receiving IV FCM at a mean dose of 1139 mg, 63 percent of the patients achieved the primary efficacy endpoint, which is a rise of ≥2g/dL or hemoglobin normalization [6].

Some studies have demonstrated that even a lower dose of 500 mg of FCM can result in significant improvement in patients. Siqueira et al. carried out a retrospective observational cohort analysis on 25 Crohn's disease patients, highlighting the importance of FCM as a treatment strategy. After receiving a single dosage of 500 mg IV FCM, they observed raised iron tests (serum iron, ferritin, and TSAT), increased Hb levels (93% of patients), and a significant improvement in hematocrit (88% of patients). Its superiority over iron hydroxide sucrose administration in the treatment of patients with refractory IDA was shown by this investigation [12]. Another study conducted by Beigel et al. revealed that over the follow-up period, 90.7 percent of the patients achieved Hb levels > 12/13 g/dl (p < 0.001). This study found that 500 mg of FCM once was sufficient to bring Hb levels back to normal in the majority of iron-deficient IBD patients [19]. 

While the majority of studies indicate that IV iron is more efficient than oral iron in treating IDA in patients suffering from IBD, a few studies in our review demonstrated comparable outcomes between the IV and oral iron groups. Bertani et al. conducted a prospective, randomized, open-label study in 40 UC patients in remission over 12 weeks, where half of them received IV FCM and the other half received oral sucrosomial iron (SI). Their study found no significant treatment differences (treatment effect (p = 0.25); interaction effect between treatment and time (p = 0.26).) between the two groups. Both treatments were well tolerated, and the study suggests the use of oral SI in the patients of UC in remission [25]. Another study conducted by Howaldt et al. 2022 was an open-label, phase three-b trial that compared IV FCM and ferric maltol for the therapy of IDA in IBD patients. For the key Hb responder rate criterion at week 12, because ferric maltol took longer to elevate Hb than IV FCM did, ferric maltol did not demonstrate noninferiority to IV FCM. But it's noteworthy that after 12 weeks, Hb levels elevated in both groups, which was clinically significant. The mean hemoglobin increases were 2.5 g/dL for ferric maltol and 3.0 g/dL for FCM. In line with IV iron, ferric maltol demonstrated similar long-term efficiency in sustaining Hb increases and raising ferritin to week 52. This study concludes that ferric maltol offers simple extended care for chronic IDA in IBD patients [4]. While some studies [4,25] have suggested that oral iron formulations may offer similar efficacy to IV iron in mild cases of IDA in IBD, FCM remains superior in more severe cases, particularly when rapid iron replenishment is required.

Studies have also revealed a correlation between the degree of activity of this disease and decreased hemoglobin levels [34,35]. Sobrado et al. conducted a study where, among 10 patients suffering from Crohn's disease who were administered an average dose of 1650 mg of IV ferric carboxymaltose, anemia normalization occurred in 70% of the patients. The current investigation demonstrates a significant correlation between the degree of CD activity as assessed by C-reactive protein (CRP) and Crohn's disease activity index (CDAI) and the severity of anemia. The three patients who required hospitalization had CDAI scores exceeding 250, and all of them had Hb levels below 8.5g/dL [21].

Observational, non-interventional prospective pilot examinations have been done by Cortes et al. to analyze the effectiveness and safety of FCM administered right after administering Infliximab (IFX). For each of these efficacy indicators, there were no statistically significant differences among the control as well as one-time therapy groups. Their study showed FCM given in one session following IFX to be safe, effective, and have a good cost-benefit ratio in IBD patients with an increased therapy adherence [20]. Nevertheless, more controlled prospective trials with a bigger sample size are necessary.

Evstatiev et al. in 2011 carried out a multicenter, controlled, randomized, open-label research study with 485 patients. The patients were given 200 milligrams of iron infusions up to 11 times using Ganzoni-calculated iron sucrose dosages, or FCM, in a maximum of three infusions of 1000/500 mg iron. This study showed that a non-anemic state or an Hb rise of ≥2g/dL was more likely to be achieved by the FCM group than by the IS group (83.8% vs. 75.9% of patients). This study showed the superiority of FCM over Ganzoni-calculated IS dosage [23]. Afterward, non-anemic patients who had finished the FERGIcor study participated in a single-blind, multicenter investigation (Evstatiev et al. 2011) conducted by Evstatiev et al. 2012, where the participants received either IV FCM 1000 mg or placebo. Kaplan-Meier analysis showed patients receiving FCM had a significantly lower chance of developing anemia than those receiving a placebo (27.2% vs 40.4%). The study concluded that patients whose anemia has responded to intravenous iron replacement therapy in the past could benefit from safe and efficient treatment with serum ferritin-triggered therapy with FCM [24].

Safety Outcomes of Ferric Carboxymaltose

In clinical trials, FCM was well tolerated by patients with iron-deficiency anemia; the majority of adverse effects associated with the treatment were deemed to be mild to moderate in intensity. In our review, the five most common adverse effects reported were nasopharyngitis, hypophosphatemia, elevated transaminases, vitamin D deficiency, and headache. Only one serious side effect, pulmonary embolism, was reported in a single patient in the study conducted by Evstatiev et al. (2011) [23]. Other common adverse effects included nausea, abdominal pain, arthralgia, rash, dizziness, constipation, diarrhea, and injection-site reactions [36]. FCM has a generally favorable safety profile. Nonetheless, hypophosphatemia brought on by iron is a well-known adverse impact.

Hypophosphatemia With Ferric Carboxymaltose

Some studies have reported high rates of hypophosphatemia following FCM administration, which, in severe cases, can result in complications like osteomalacia and fractures. According to research, FCM causes an acute increase in blood levels of physiologically active fibroblast growth factor 23, which in turn causes a rise in urine phosphate excretion and a decrease in serum levels of 1,25-dihydroxyvitamin D, leading to a high prevalence of hypophosphatemia [37,38]. Although the heart, liver, thyroid, and parathyroid glands all contain FGF23, the bone is the main source of its synthesis. It activates FGFR/αKL receptor complexes in renal tubules to impede phosphate reabsorption and also reduce activation of 1,25-dihydroxy vitamin D [39]. The relationship between the metabolism of phosphorus and iron administered intravenously was first observed by Okada et al. in 1983. Within two weeks of starting saccharated iron oxide, seven out of nine patients had developed hypophosphatemia [40]. Severe hypophosphatemia can cause serious adverse effects such as heart failure, pulmonary failure, and rhabdomyolysis. Fractures and osteomalacia can exacerbate long-term hypophosphatemia [41].

A prospective seven-week observational study by Detlie et al. compared the incidence of hypophosphatemia between the patients receiving a 1000 mg dose of either FCM or ferric derisomaltose. At week six, hypophosphatemia was noted in 3.7 percent of patients receiving the FDI and in 21.6 percent of patients who had started FCM therapy. Furthermore, there was a significant high in the mean urine fractional excretion of phosphate in patients receiving FCM, as well as significantly greater mean concentrations of intact fibroblast growth factor 23 (P < 0.001). The investigation came to the conclusion that FCM-induced hypophosphatemia is largely caused by fibroblast growth factor 23 [22].

An RCT was conducted by Zoller et al. comparing the hypophosphatemia seen in patients receiving a 1000 mg dose of either FCM or ferric derisomaltose. Patients treated with FCM experienced hypophosphatemia more frequently than those treated with FDI at every visit; the prevalence peaked at 45.8% two weeks after the first FCM medication. After both FCM infusions, intact FGF23 concentrations were markedly elevated in comparison to FDI. Patients receiving FCM had higher urinary phosphate excretion [26].

The degree of hypophosphatemia determines the course of treatment; oral or intravenous phosphate substitution is usually necessary for most individuals. Potential future treatment strategies may include the use of therapeutic anti-FGF23 antibodies, such as KRN23 [41].

This review has limitations, many of which are common to indirect comparisons of interventions and systematic searches. First, the selection of only three databases may have introduced selection bias. Additionally, the trials showed variations in treatment duration, dosages, and clinical outcomes. Baseline hemoglobin values and hematological inclusion criteria were not uniformly distributed across studies. Furthermore, some trials did not align with our primary outcome measure of response, defined as either a ≥2 g/dL increase in hemoglobin or hemoglobin normalization. Furthermore, the scope of our investigation required reliance on published study reports rather than raw data, limiting our ability to control for heterogeneity and potential reporting bias.

## Conclusions

In conclusion, this systematic review underscores the effectiveness and safety of intravenous FCM as a viable treatment option for IDA in adults with inflammatory bowel disease (IBD).The findings consistently demonstrate that FCM is among the most efficacious intravenous iron therapies, leading to significant improvements in hemoglobin levels and other iron profile parameters in this patient population. This makes FCM a favorable option for rapidly correcting iron deficiency along with anemia in IBD patients, who often struggle with poor absorption of oral iron due to underlying disease pathology. However, from a safety perspective, FCM is notably associated with the risk of hypophosphatemia, which could have clinical consequences, especially with long-term use. This adverse effect should be closely monitored in patients receiving FCM, and healthcare providers should be aware of its potential risks.

Several important concerns still warrant further investigation, particularly the long-term security of administering high doses of IV iron, especially its effects on phosphate metabolism and other potential adverse outcomes. Additionally, the effectiveness of maintenance therapy in sustaining normal ferritin and hemoglobin levels, along with its impact on patient quality of life, requires more comprehensive research.
